# A Patient With Brain Metastasis From a Gastric Tumor

**DOI:** 10.7759/cureus.89888

**Published:** 2025-08-12

**Authors:** Gerald J Vega Castillo, Christopher Kaleb Romero Ríos, Jasson E Rostran, Jairo Rodolfo Moreno Tova, Evelyn Shirley Chavez Centeno, Luis M Polanco

**Affiliations:** 1 Oncology, Hospital Militar Escuela "Dr. Alejandro Dávila Bolaños", Managua, NIC; 2 School of Medicine, Hospital Militar Escuela "Dr. Alejandro Dávila Bolaños", Managua, NIC; 3 Research, Hospital Militar Escuela "Dr. Alejandro Dávila Bolaños", Managua, NIC; 4 Pathology, Hospital Militar Escuela "Dr. Alejandro Dávila Bolaños", Managua, NIC

**Keywords:** adenocarcinoma, brain metastasis, chemotherapy, gastric cancer, her2, neurosurgery

## Abstract

Brain metastases secondary to gastric carcinoma are exceedingly rare and are associated with a poor prognosis. Their clinical presentation may mimic common neurological conditions, making early diagnosis and timely management challenging. We report the case of a 68-year-old male with a history of diabetes and hypertension who presented with sudden left-sided hemiparesis and urinary incontinence. Brain CT revealed a right frontal lesion with surrounding edema. He underwent craniotomy with tumor resection, and histopathological analysis confirmed a poorly differentiated gastric adenocarcinoma with human epidermal growth factor receptor 2 (HER2) overexpression (3+), consistent with brain metastasis of gastric origin. Systemic treatment with oxaliplatin and capecitabine was initiated, and anti-HER2-targeted therapy was considered. This report highlights the importance of maintaining a high index of suspicion for brain metastasis in patients with space-occupying lesions, even in the absence of a prior cancer diagnosis. The combination of neuroimaging, surgery, immunohistochemistry, and tailored oncologic treatment enabled timely intervention. The report underscores the value of interdisciplinary management and the clinical utility of biomarkers such as HER2 in guiding therapy in rare clinical scenarios.

## Introduction

Upper gastrointestinal tract malignancies, including gastric cancer, are common worldwide. Gastric cancer ranks fourth to fifth in global cancer incidence and is among the top three causes of cancer-related mortality [1]. The common metastatic sites include the liver, lungs, and bones; however, central nervous system (CNS) dissemination occurs in less than 1% of cases, significantly impacting prognosis and management [2]. Patients with brain metastases from gastric tumors have a median overall survival of less than one year, worse than that seen with other cancers such as melanoma, breast, or lung cancer [3]. Recent advancements in treatment include surgery for localized disease and chemotherapy, radiotherapy, and targeted therapies for advanced stages. Nevertheless, due to the rarity of CNS involvement, no standardized treatment protocol currently exists.

## Case presentation

A 68-year-old male with a history of type 2 diabetes mellitus and systemic arterial hypertension presented to the emergency department following an acute neurological event that had occurred earlier that morning. While attempting to rise from bed, he had suddenly collapsed due to left-sided weakness and experienced urinary incontinence. On arrival, his vital signs were as follows: blood pressure: 120/75 mmHg, mean arterial pressure: 90 mmHg, heart rate: 114 beats per minute, respiratory rate: 17 breaths per minute, oxygen saturation: 96%, axillary temperature: 38 °C, and capillary glucose: 114 mg/dL. Neurological examination revealed left-sided hemiparesis, with muscle strength graded 3/5 according to the Medical Research Council (MRC) scale in both upper and lower extremities [3]. There was no evidence of aphasia, mental status changes, or altered consciousness. His National Institutes of Health Stroke Scale (NIHSS) score was 8, indicating a moderately severe neurological event [3].

**Figure 1:**
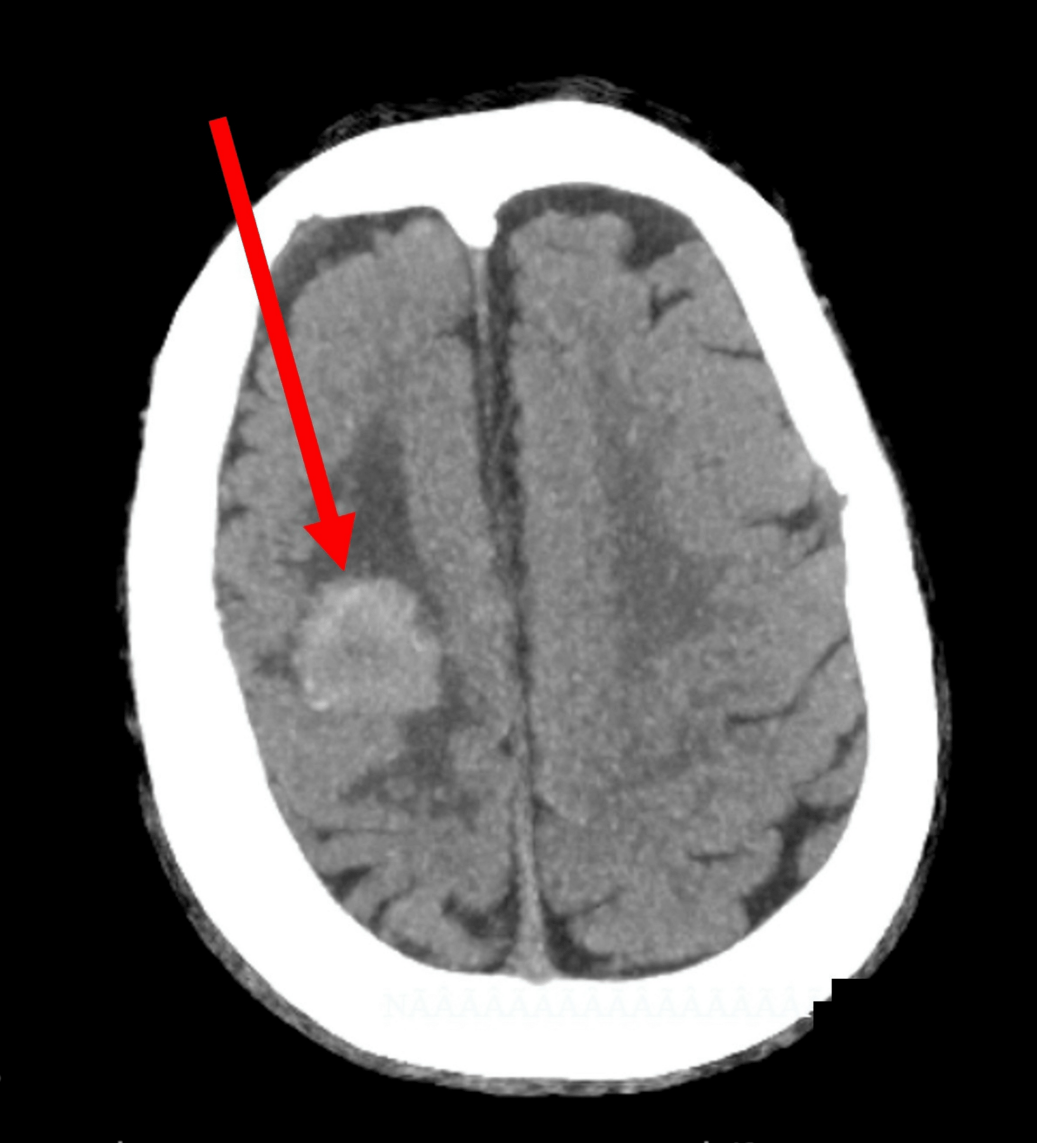
Axial brain CT scan The red arrow indicates an intra-axial lesion seen in the right frontal lobe, located at the precentral gyrus, oval-shaped, and isodense to gray matter, surrounded by vasogenic perilesional edema. It measures 26 x 20 mm in maximum diameter and is associated with a mass effect, partially compressing the right lateral ventricle CT: computed tomography

**Figure 2:**
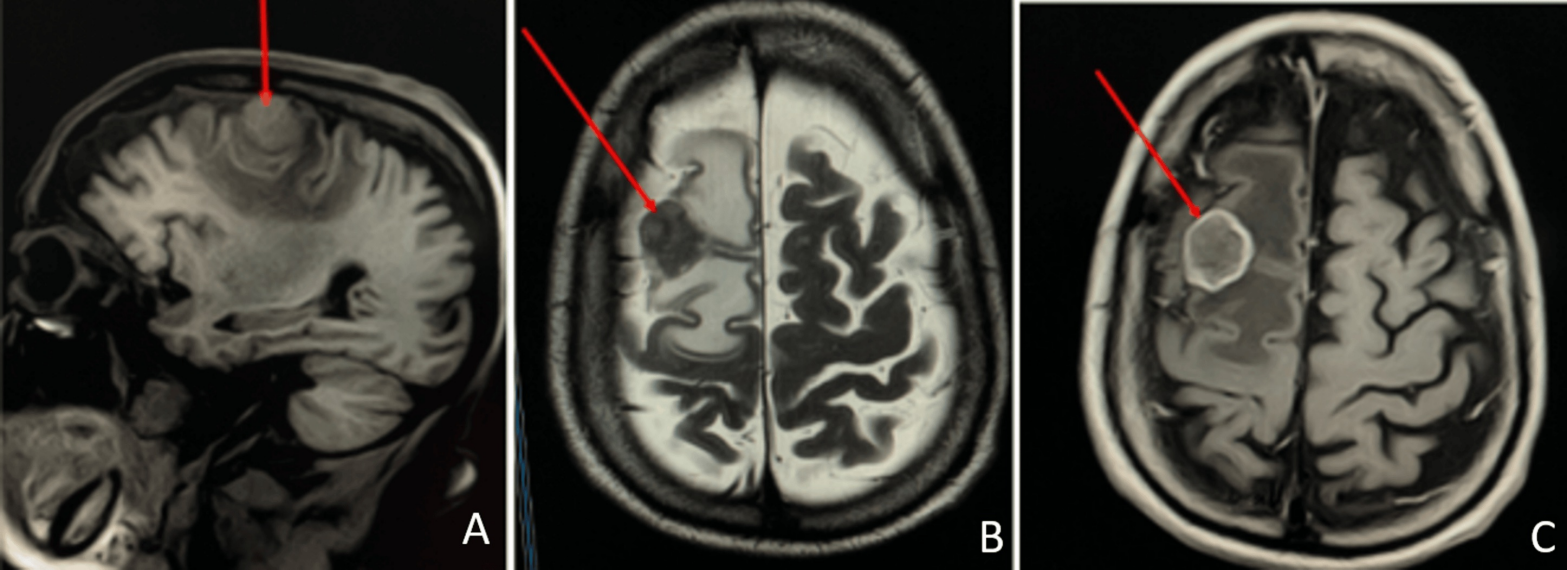
Brain MRI (A) Sagittal view showing a well-defined nodular lesion (red arrow) observed in the precentral gyrus of the right frontal lobe, with signal intensity isointense to the gray matter. (B) Axial T1-weighted image without contrast, confirming the isointense signal of the lesion relative to the cerebral cortex; red arrow shows the lesion. (C) Axial T2-weighted image; the red arrow shows the hypointensity of the lesion, surrounded by a broad area of hyperintensity consistent with vasogenic edema, causing effacement of adjacent sulci and fissures. Postcontrast sequences demonstrate peripheral ring enhancement and restricted diffusion, suggestive of residual tumor. The lesion measures approximately 19 × 16 mm in the axial plane MRI: magnetic resonance imaging

An urgent head CT scan (Figure 1) identified a right frontal mass lesion with surrounding vasogenic edema and resulting mass effect; no acute intracranial hemorrhage was noted. There were no CT signs suggestive of acute ischemic infarction apart from the mass lesion. Subsequently, brain MRI (Figure 2) demonstrated a well-defined, heterogeneously enhancing mass in the right frontal lobe with pronounced perilesional edema and midline shift. Diffusion-weighted imaging showed no restricted diffusion, effectively excluding acute ischemic infarction as the primary cause of his neurological presentation. No additional intracranial lesions were observed.

Further inquiry revealed a four-month history of progressive dysphagia and an approximately 32-kg unintentional weight loss. Outpatient upper gastrointestinal endoscopy performed weeks prior had revealed a Bormann type IV gastric tumor, from which biopsy samples had been taken. The patient remained unaware of the biopsy findings due to the family’s decision to await definitive pathology confirmation.

**Figure 3:**
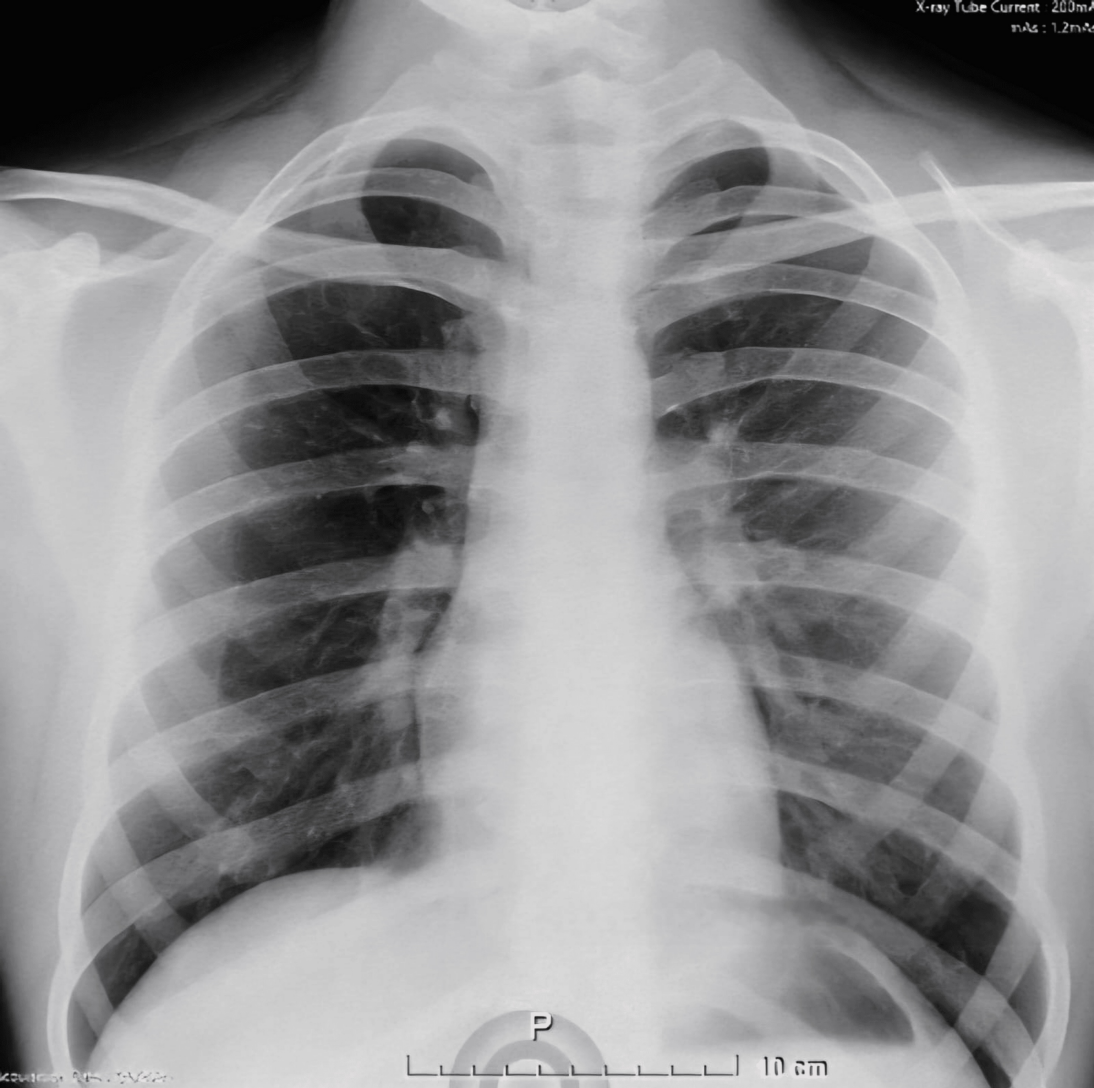
Chest X-ray The lungs are well aerated with no evidence of infiltrates, consolidations, masses, or pleural effusion. The mediastinum and vascular structures are within normal limits. The diaphragm has sharp contours with no elevation or flattening. Costophrenic and cardiophrenic angles are clear. Bony structures show no apparent abnormalities

In the 24 hours preceding admission, he had experienced persistent fever unresponsive to a single dose of acetaminophen. He also reported a three-month history of intermittent cough, initially productive of whitish sputum that had later turned yellow without associated dyspnea. A chest radiograph (Figure 3) showed no consolidation or mass lesion. A fever workup in the emergency department - including blood cultures, inflammatory markers, and further imaging - was initiated to identify the etiology.

Given the neurological deficits and imaging findings, the patient underwent craniotomy with microsurgical resection of the right frontal tumor, performed under intraoperative cortical and subcortical neuromonitoring along with neuronavigation. Postoperatively, he was transferred to the coronary care unit and received cefazolin, dexamethasone, and supportive care. Initial recovery was satisfactory, and a follow-up head CT revealed no acute surgical complications.

**Figure 4:**
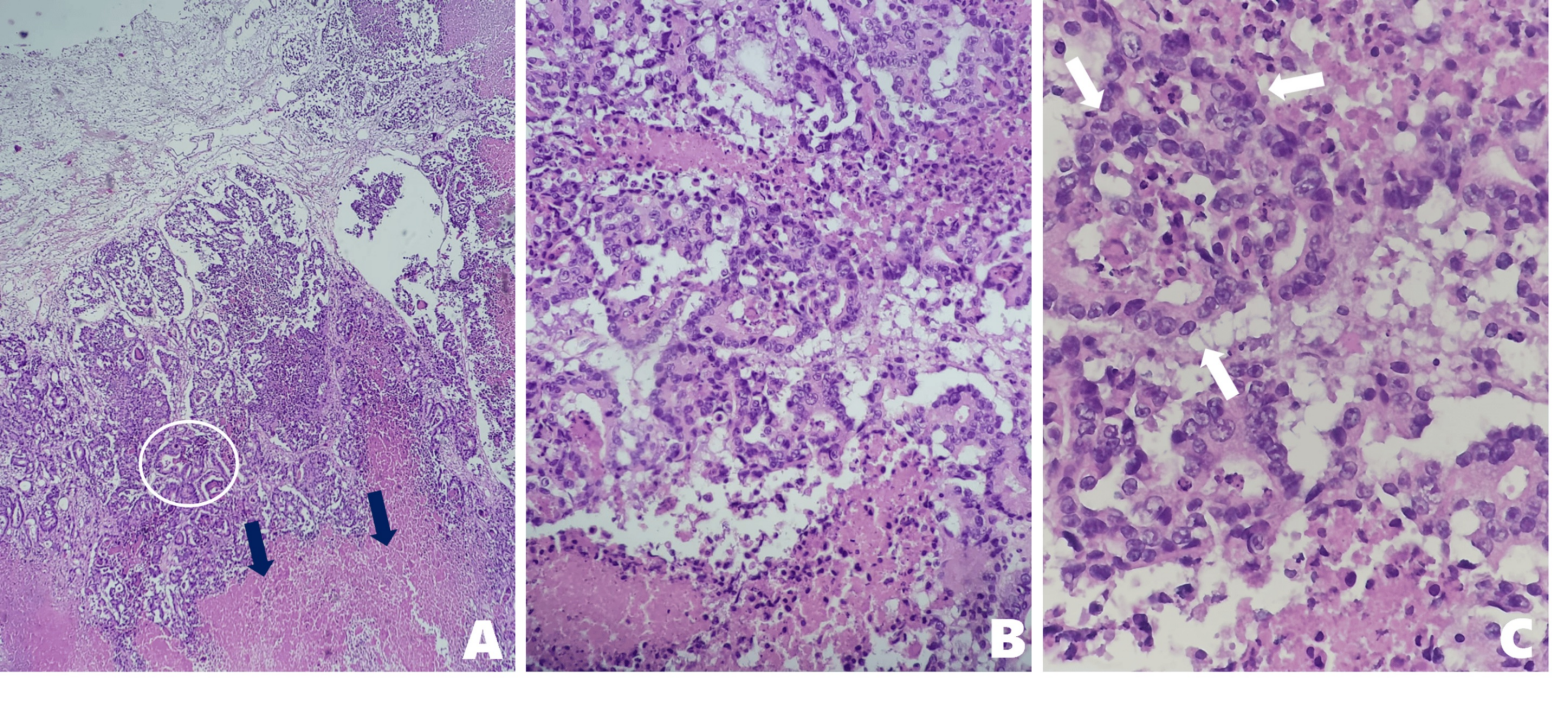
Histopathological examination with H&E staining of brain metastasis from gastric adenocarcinoma (A) Low magnification (4×): Cerebral tissue infiltrated by tumor; areas of necrosis (amorphous eosinophilic masses) and pseudoglandular formations are observed, disrupting the normal architecture. (B) Intermediate magnification (10×): Disorganized proliferation of tumor cells forming irregular pseudoglands, with pleomorphic and hyperchromatic nuclei. (C) High magnification (40×): Cells of varying sizes and shapes, with prominent nucleoli and marked basophilia, indicating high mitotic activity

**Figure 5:**
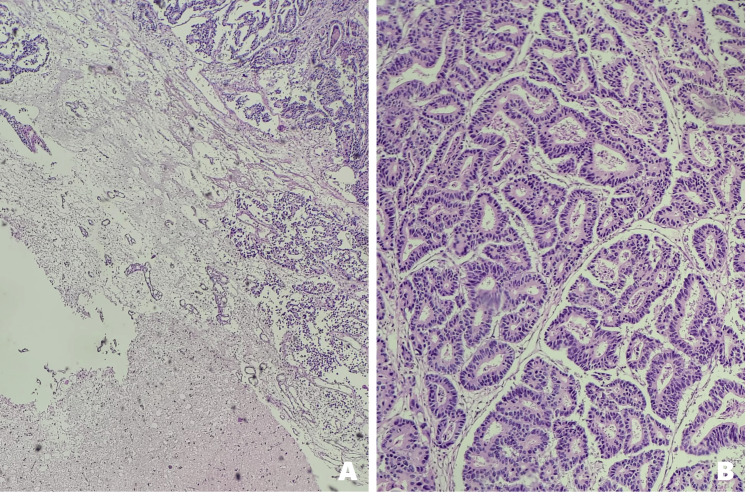
Histological morphology of brain metastasis from gastric adenocarcinoma (A) Low magnification (4×): Partially preserved cerebral parenchyma adjacent to tumor regions showing complete loss of normal architecture and the presence of irregular pseudoglandular structures. (B) Higher magnification (10×): Tumor cells with basophilic nuclei, dense chromatin, prominent nucleoli, and frequent mitotic figures, arranged in atypical pseudoglands, confirming metastatic infiltration from gastric adenocarcinoma

**Figure 6:**
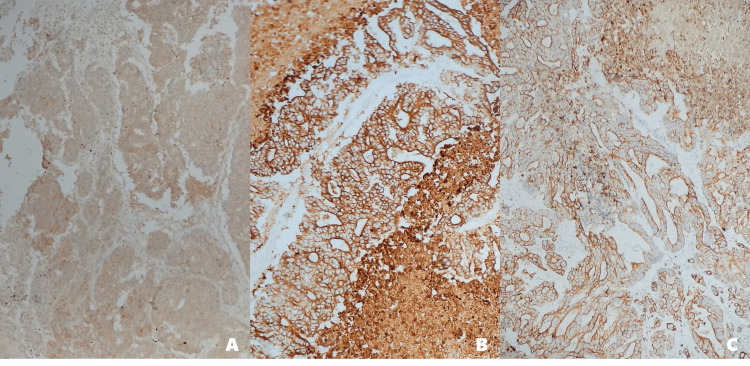
Immunohistochemistry of brain tissue with metastatic gastric adenocarcinoma (A) HER2-positive (+++): Strong membranous staining in tumor cells, consistent with overexpression (score 3+). (B) Cytokeratin 7 (CK7)-positive (+++): Diffuse cytoplasmic staining, indicative of glandular epithelial differentiation. (C) Cytokeratin 19 (CK19) positive (+++): Supports the epithelial origin of the tumor. The immunophenotypic profile is consistent with metastatic HER2-positive gastric adenocarcinoma HER2: human epidermal growth factor receptor 2

Histopathological analysis of the resected lesion (Figure 4) revealed a poorly differentiated adenocarcinoma, confirming metastatic gastric carcinoma (Figure 5). Immunohistochemical staining (Figure 6) showed strong diffuse positivity for cytokeratin 7 (CK7), cytokeratin 19 (CK19), and human epidermal growth factor receptor 2 (HER2/neu) with a score of 3+. HER2 status was reassessed in the metastatic brain specimen to confirm concordance with the primary tumor, acknowledging known intratumoral and intertumoral heterogeneity in HER2 expression in gastric carcinoma, which has significant treatment implications.

Following neurosurgical recovery, the patient was evaluated by oncology and initiated on systemic chemotherapy comprising oxaliplatin administered intravenously on day 1 of each cycle, and capecitabine given orally twice daily from days 2 to 15. Treatment cycles were scheduled every three weeks for a total of eight cycles. Given the confirmed HER2 overexpression, the patient was also considered a candidate for targeted therapy with trastuzumab, which is known to improve outcomes in HER2-positive advanced gastric carcinoma. Family counseling was provided, and follow-up appointments were arranged with both oncology and neurosurgery.

## Discussion

According to GLOBOCAN 2020, there were 1,089,000 new cases of gastric cancer worldwide, with an age-standardized incidence rate of 11.1 per 100,000, making it the fifth most common malignancy. In the same year, it accounted for 769,000 deaths (age-standardized mortality rate: 7.7 per 100,000), ranking fourth in cancer-related mortality, behind lung, colorectal, and liver cancers. Mortality rates vary by country [4]. Gastric cancer is a multifactorial disease. Helicobacter pylori infection is the main risk factor, though only 17% of those infected develop cancer. Incidence is two to three times higher in men and increases significantly after the age of 65 years. Other risk factors include smoking, alcohol consumption (both classified by the International Agency for Research on Cancer (IARC) as Group 1 carcinogens), metabolic dysfunction, and elevated BMI (>25) [5].

Brain metastasis as the initial presentation of gastric adenocarcinoma is extremely rare. Incidence in gastric cancer patients ranges from 2 to 3%, but may be higher (up to 8-24%) in those with HER2 overexpression [6,7]. Symptoms are often nonspecific and may mimic other acute neurological events such as stroke, with manifestations including hemiparesis, headache, seizures, altered mental status, or signs of intracranial hypertension. In other cases, metastases are incidental findings on neuroimaging [7,8].

Our patient presented with sudden hemiparesis and urinary incontinence in the setting of cardiovascular risk factors, initially suggesting stroke. Brain CT revealed a space-occupying lesion with edema, prompting MRI, which confirmed a right frontal tumor suggestive of metastasis. Unlike stroke, metastatic brain lesions typically appear as well-defined nodules with contrast enhancement and surrounding vasogenic edema on imaging [8,9]. Neurosurgical resection was deemed appropriate given the solitary, accessible lesion with mass effect. Surgery confirmed the diagnosis and provided therapeutic benefit. Resection of single brain metastases in patients with good functional status and controlled extracranial disease has been associated with improved survival compared to radiotherapy or palliative care alone [7,8].

Immunohistochemistry was crucial in identifying the tumor origin. In this case, strong CK7/CK19 positivity and HER2 overexpression confirmed a metastatic gastric adenocarcinoma [8-10]. Immunohistochemistry is indispensable in cases of brain metastases from unknown primaries, as it informs systemic therapy decisions. HER2 status has therapeutic implications. Although HER2 overexpression is associated with increased brain metastases, it may also confer a better prognosis due to the availability of targeted therapies. Our patient received oxaliplatin-capecitabine chemotherapy while awaiting further evaluation for anti-HER2 therapy [8-10]. In advanced gastric and gastroesophageal adenocarcinomas, this regimen is a preferred first-line option, offering efficacy comparable or superior to fluorouracil-cisplatin combinations, with better toxicity profiles, especially reduced nephrotoxicity and neutropenia [10-12].

Although trastuzumab has limited CNS penetration, there are reports of clinical benefit when combined with surgery or radiotherapy in selected patients. New anti-HER2 agents with improved CNS penetration are under investigation, though no standard regimen currently exists for HER2+ brain metastases in gastric cancer [5,13].

## Conclusions

This report highlights a rare and clinically significant presentation of advanced gastric adenocarcinoma, where the initial manifestation was an acute neurological event: hemiparesis due to a solitary brain metastasis. CNS involvement from gastric cancer is exceedingly rare and poses major diagnostic and therapeutic challenges when it appears as the first symptom. The significance of this case lies not only in the rarity of the presentation but in the opportunity it provided to establish the primary cancer diagnosis, perform surgical resection of the brain lesion, and initiate personalized systemic treatment guided by immunohistochemistry, including HER2 positivity. Furthermore, the report emphasizes the importance of a comprehensive and coordinated clinical approach to acute neurological symptoms in patients with unknown or pending cancer diagnoses. The integration of advanced neuroimaging, histopathology, and immunohistochemistry was key to accurate etiological diagnosis. Overall, this report reinforces the need to maintain high clinical suspicion for atypical brain lesions and always consider metastases from visceral tumors not typically associated with CNS involvement, such as gastric cancer.
